# Combination of IL-2, rapamycin, DNA methyltransferase and histone deacetylase inhibitors for the expansion of human regulatory T cells

**DOI:** 10.18632/oncotarget.10914

**Published:** 2016-07-28

**Authors:** Makoto Miyara, Driss Chader, Aude Burlion, Jérémie Goldstein, Delphine Sterlin, Françoise Norol, Hélène Trebeden-Nègre, Laetitia Claër, Shimon Sakaguchi, Gilles Marodon, Zahir Amoura, Guy Gorochov

**Affiliations:** ^1^ Department of immunology, AP-HP Pitié Salpêtrière, Paris, France; ^2^ Sorbonne Universités, UPMC Univ Paris 06, INSERM, CNRS, Centre d'Immunologie et des Maladies Infectieuses (CIMI-Paris), Paris, France; ^3^ Cell Therapy, AP-HP Pitié Salpêtrière, Paris, France; ^4^ Experimental Immunology, Immunology Frontier Research Center, Osaka University, Osaka, Japan; ^5^ Internal Medicine, French Reference Center for Systemic Lupus Erythematosus and Antiphospholipid Syndrome, AP-HP Pitié Salpêtrière, Paris, France; ^6^ UPMC Paris Sorbonne, Paris, France

**Keywords:** human regulatory T cells, FOXP3, cell therapy, autoimmunity, GVH

## Abstract

FOXP3^+^ regulatory T cell (Treg) based cellular therapies represent promising therapeutic options in autoimmunity, allergy, transplantation and prevention of Graft Versus Host (GVH) Disease. Among human FOXP3-expressing CD4^+^T cells, only the CD45RA^+^ naïve Treg (nTreg) subset is suitable for *in vitro* expansion. However, FoxP3 expression decays in cells using currently described culture protocols.

Rapamycin alone was not able to prevent FOXP3 loss in nTregs cells, as only a half of them maintained FOXP3 expression after 14 days of culture. In contrast we report a novel combined drug regimen that can drastically stabilize FOXP3 expression in cultured Tregs. IL-2, rapamycin, histone deacetylase and DNA methyltransferase inhibitors act in synergy to allow expansion of human regulatory T cells with sustained high expression of FOXP3 and CD15s with potent suppressive capacities *in vitro* and control of murine xeno-GVH reactions. Of note, an additional subsequent infusion of expanded nTreg cells did not improve survival of mice.

Combination of IL-2, rapamycin, histone deacetylase and DNA methyltransferase inhibitors is optimal for the expansion *in vitro* of pure effective nTreg maintaining high levels of FOXP3 for therapeutic purposes.

## INTRODUCTION

Thymus derived naturally occurring FOXP3 expressing CD4^+^ regulatory T cells (Treg cells) are indispensable for the maintenance of self tolerance and immune homeostasis [1]. Treg cells play a crucial role in the prevention of autoimmune diseases, allergy and allograft rejection. Manipulation of Treg cell biology has therefore emerged as a potential alternative strategy for the control of immune responses [2].

Transfer of Treg cells isolated from spleen and lymph nodes of healthy mice have proven efficient in the prevention of numerous autoimmune diseases in various animal models [3–6], leading to the assumption that Treg cell could represent a new therapeutic option in autoimmunity [7, 8].

Because FOXP3 expressing Treg cells represent 1 to 6 % of CD4^+^ T cells in humans [9], *in vitro* expansion strategies are required to enable the infusion of significant number of Treg cells, given that 2 million Treg cells are usually required for infusion in mice (10^5^ Treg cells per gram) to prevent autoimmunity [10].

Human Treg cells were initially defined as CD4^+^CD25^high^ T cells [11–16]. Thus, most strategies aiming at the expansion of human Treg cells have been mainly based on the isolation of CD25^+^CD4^+^ T cells, giving rise to expanding cells that contain significant proportions of cells that do not express FOXP3 [17, 18]. Because FOXP3 is a key molecule in the development and function of Treg cells, and because high levels of FOXP3 are more correlated with potent suppression than low levels of FOXP3 [19], Treg expansion protocols should incorporate means to maintain high levels of FOXP3 expression.

We have previously shown that human FOXP3 expressing CD4^+^ T cells are composed of three subsets that are phenotypically and functionally distinct: CD45RA^+^FOXP3^low^ naïve Treg cells (nTreg cells) and CD45RA^−^FOXP3^high^ effector Treg cells (eTreg cells) and CD45RA^−^FOXP3^low^ non suppressive T cells (FOXP3^low^ non Treg cells) [20]. In addition, we have recently shown that, among CD45RA^−^FOXP3^+^ cells, expression of surface marker CD15s (sialyl Lewis x) could differentiate eTreg cells, that are CD15s^+^, from FOXP3^low^ non Treg cells that do not express CD15s [21]. Because FOXP3 expressing CD4^+^ T cells are heterogeneous, it is necessary to study FOXP3 and suppressive capacities of each *in vitro*-expanded subset. It is also important to determine which pathways can be manipulated in order to enhance FOXP3 expression and maintain suppressive capacities in cultured Treg cells. Currently accepted reference protocol for the *in vitro* expansion of human Treg cells is based on the use of rapamycin in combination with IL-2 [22]. Epigenetic changes such as DNA methylation of FOXP3 genes and acetylation of histones and of the FOXP3 protein itself have been shown to be important for the stability and the suppressive function of Treg cells [23–27]. We therefore questioned whether molecules modifying the epigenetics of Treg cells could enhance their expression of FOXP3 and/or their suppressive capacities *in vitro* or *in vivo* and whether their effects were better than the ones observed with rapamycin.

Here we report a novel combined drug regimen that can drastically stabilize FOXP3 expression in cultured Treg cells. IL-2, rapamycin, histone deacetylase and DNA methyltransferase inhibitors act in synergy to allow expansion of human regulatory T cells with sustained high expression of FOXP3 and CD15s with potent control of murine xeno-Graft *Versus* Host (GVH) reactions.

## RESULTS

### IL-2/rapamycin combination partially maintains FOXP3 expression in expanding FOXP3^+^ CD4^+^ T cell subsets

FOXP3 expressing CD4^+^ T cells are heterogeneous in terms of FOXP3 expression levels and suppressive capacities [9]. We examined whether purified FOXP3-expressing CD4^+^ T cells subsets had different fates upon expansion *in vitro*.

**Figure 1:**
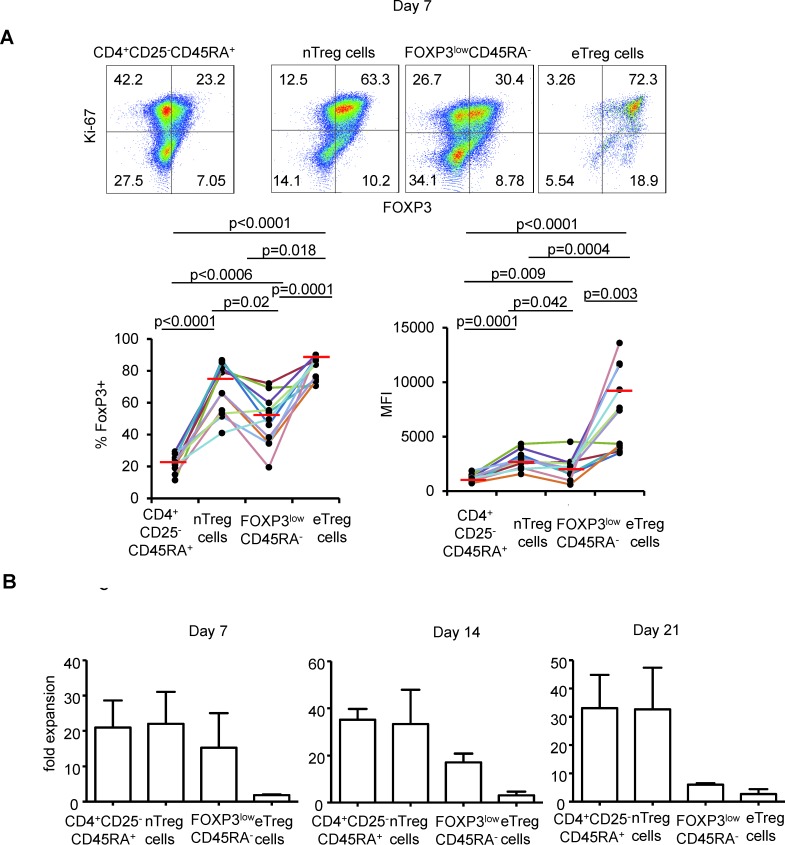
FOXP3 expression following expansion in the presence of high dose IL-2 **A.** CD25^−^CD45RA^+^CD4^+^ T cells, nTreg cells, FOXPp3^low^ non Treg cells and eTreg cells were flow isolated according to the gating strategy described in Supplementary Figure 1 and cultured for 7 days in the presence of anti-CD3/CD28 beads and IL-2 and analyzed for FOXP3 and Ki-67 expression. Representative flow cytometry of indicated CD4^+^FOXP3 expressing subsets from 11 individual experiments is shown (top). Threshold for FOXP3 expression is defined in CD4^+^CD25^−^CDR45RA^+^ T cells after 7 days of culture. Numbers indicate % of cells in each quadrant. Proportions of FOXP3^+^ cells and MFI corresponding to FOXP3 expression of indicated CD4^+^FOXP3 expressing subsets for each individual experiment are presented using distinct colors (*n* = 11, bottom). Red horizontal bars represent mean percentages. Comparisons were made using the Wilcoxon matched pairs test. **B.** Fold expansion obtained after 7, 14 and 21 days of culture in the presence of anti-CD3/CD28 beads and IL-2 by indicated CD4^+^FOXP3 expressing subsets in 3 independent experiments. Error bar represent s.d.

Neither FOXP3^low^ CD45RA^+^nTreg nor FOXP3^low^CD45RA^−^ non Treg cells were able to maintain FOXP3 expression in culture. In contrast eTreg cells maintained high levels of FOXP3 expression under the same culture conditions in term of percentage of cells and of MFI. However, the latter cells were poorly proliferative even in the presence of high dose IL-2 (Figure 1).

**Figure 2:**
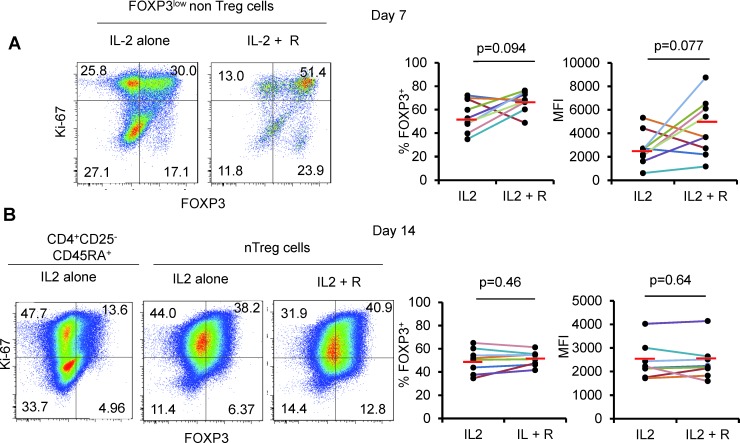
Rapamycin does not prevent FOXP3 downregulation **A.** FOXP3^low^ non Treg cells and (B) CD25^−^CD45RA^+^CD4^+^ T cells and nTreg cells were isolated and cultured for 14 days as in Figure 1 with or without addition of rapamycin (R) and then analyzed for FOXP3 and Ki-67 expression. Threshold for FOXP3 expression is defined in CD4^+^CD25^−^CDR45RA^+^ T cells after 14 days of culture. Representative Flow cytometry data (left) and summary of 8 color-coded individual experiments (right). Red horizontal bars represent mean percentages. Comparisons were made using the Wilcoxon matched pairs test.

We also monitored longitudinally FOXP3^low^nonTreg cells and observed that about a half of expanding cells maintained FOXP3 expression upon expansion *in vitro* (mean % +/−SD: 55 +/− 13.5, mean MFI+/−SD: 2682 +/− 1416 after 7 days of culture). This indicates that some FOXP3^low^ CD45RA^−^ cells may have eTreg differentiation potentiality. In the presence of rapamycin in addition to IL-2, the proportion of expanding FOXP3^low^CD45RA^−^ non Treg cells maintaining FOXP3 expression was higher (mean % +/−SD: 66.9 +/− 8.4, mean MFI +/− SD: 4466 +/− 2411), which is consistent with the finding that rapamycin promotes the expansion of genuine Treg cells at the expense of non Treg cells [22]. However, significant proportion of expanding non Treg cells still lost FOXP3 expression after 7 days of culture, even in the presence of rapamycin (Figure 2A). This finding in addition to the poor capacity of eTreg cells to survive even in the presence of high dose IL-2 indicates that FOXP3^+^ cells with a CD45RA^−^ phenotype are improper for expansion.

In contrast to eTreg cells, naïve Treg cells were highly proliferative in the presence of IL-2 and most expanding nTreg cells maintained FOXP3 expression until day 7 (Figure 1) indicating that nTreg cells are, as previously shown by Hoffman et al., better suited to *in vitro* expansion than other FOXP3 expressing CD4^+^ T cell subsets [28]. However, expanded nTreg FOXP3 levels were clearly lower than those of expanded eTreg cells in term of % and of MFI (Figure 1). Moreover, nTreg cells progressively lost FOXP3 expression during the second week of expansion, even in the presence of rapamycin (mean % +/−SD: 49.69 +/− 10.6 and 51.6 +/− 6.26 in the presence of IL-2 alone or IL-2 and rapamycin respectively). These results indicate that neither IL-2 nor IL-2/rapamycin association can efficiently maintain FOXP3 expression in nTreg cells. (Figure 2)

### Combination of azacytidin, vorinostat, rapamycin and IL-2 enhances FOXP3 expression in expanding nTreg cells

Since the addition of rapamycin to IL-2 is insufficient to maintain high expression of FOXP3 in nTreg, we investigated which pathways could be manipulated in order to maintain FOXP3 expression upon *in vitro* expansion. Because epigenetic changes such as DNA methylation of FOXP3 genes and acetylation of histones and of the FOXP3 protein itself have been shown to participate in Treg cell stability and suppressive function [23–27], we assessed DNA methyl transferase inhibitor azacytidin and histone deacetylase inhibitor vorinostat for their capacity to enhance or even induce FOXP3 expression *in vitro*. Introduction of each molecule was by itself associated with increased proportions of FOXP3 expressing CD4^+^ T cells after 3 days of culture, compared to cells cultured in the presence of anti-CD3/anti-CD28 coated beads and high dose IL-2 alone. We also determined the optimal ranges of concentration that favor the relative expansion of FOXP3^+^ CD4^+^ T cells in such cultures without excessive toxic effects as being 1 to 10 microM for azacytidin and 0.1 to 3 microM for vorinostat. (Supplementary Figure 2)

**Figure 3:**
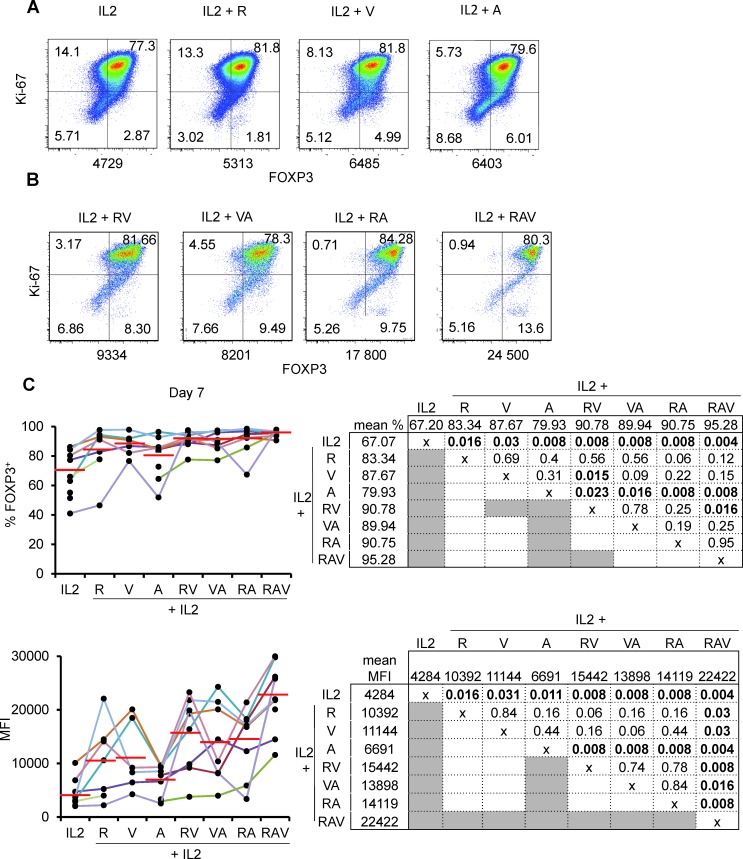
nTreg FOXP3 expression following expansion under IL-2, rapamycin, azacytidin and vorinostat combinations **A.** and **B.** Ki-67 and FOXP3 expression in nTreg isolated and cultured as described in Figure 1 with addition of (A) rapamycin (R), azacytidin (A), Vorinostat (V) indicated combinations. One representative experiment out of 8. Numbers under each panel indicate cellular MFI following anti-FOXP3 conjugated with PE. **C.** Proportions of FOXP3+ cells (top) and corresponding MFI (bottom) in expanding nTreg cells under indicated culture conditions. Each individual experiment (*n* = 8) is color-coded (left). Red horizontal bars represent mean percentages. Mean % and MFI pair wise comparisons using the Wilcoxon matched pairs test are displayed in charts (right). Significant *p* values ( < 0.05) are bold highlighted in shaded boxes.

We then compared the effects of various combinations of rapamycin, azacytidin and vorinostat on nTreg expansion in the presence of IL-2. When tested separately, azacytidin (A), vorinostat (V) and rapamycin (R) had comparable effects on cultured nTreg cells as they enhanced FOXP3 expression in terms of % and MFI. However, none of the molecules was superior to others (Figure 3A and 3C). We then combined molecules two by two [i.e. rapamycin + azacytidin (RA); rapamycin + vorinostat (RV); and vorinostat + azacytidin (VA)] and observed, at day 7 of culture, that every combination was superior to azacytidin(A) alone but not to other drugs. RV combination was also superior to vorinostat (V) alone but not to rapamycin (R) alone. Of note, none of the pair-wise combination of the molecules mentioned above was superior to others. (Figure 3B and 3C).

**Figure 4:**
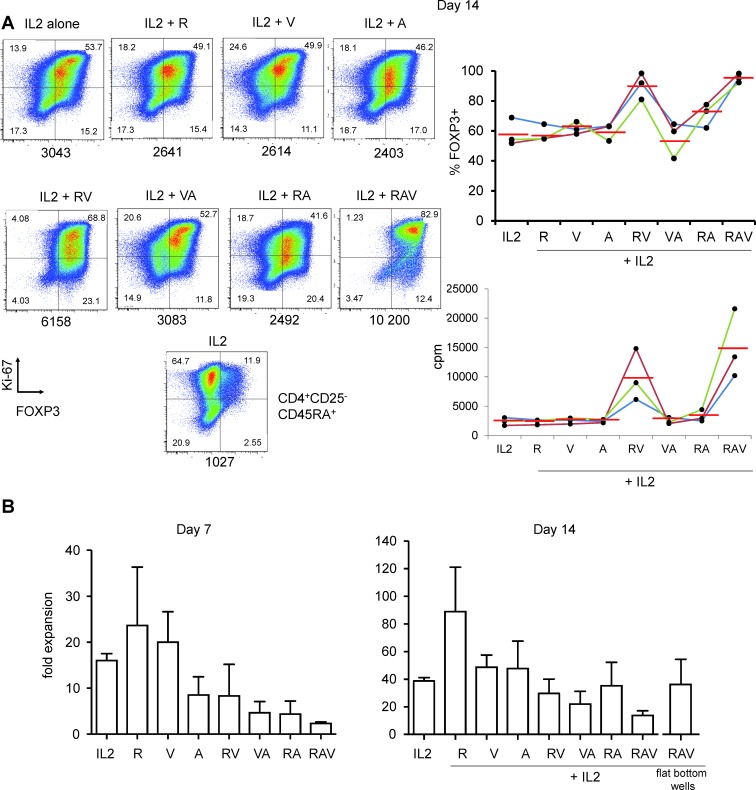
Highly pure FOXP3^high^ cells after 14 days of culture under RAV regimen **A.** Ki-67 and FOXP3 expression in nTreg cells isolated and cultured as in Figure 1, but for 14 days in the presence of indicated R, A and V combinations. Expanded CD4^+^CD25^−^CD45RA^+^ T cells were also co-analysed in order to set the threshold for FOXP3 expression (bottom plot). MFI values are indicated below each panel. One representative (left) experiment out of 3 (right). **B.** Day 7 (left) and 14 (right) expansion rate of nTreg cells in the presence of IL-2 alone or in combination with indicated molecules. Mean values with standard seviations (s.d.) from 3 independent experiments.

We thus combined rapamycin, azacytidin, vorinostat (RAV) with IL-2 and this time observed a drastic effect on the preservation and the enhancement of FOXP3 expression of cultured nTreg cells until day 14 (Figure 3B, 3C and Figure 4A). Interestingly, not only proportions of FOXP3^+^ cells, but also FOXP3 levels were increased in nTreg cells expanded in that way, compared to FOXP3 levels in nTreg cells expanded using other combinations at day 7 (see mean MFIs in Figure 3C) and day 14 of culture (Figure 4A).

We also measured nTreg cells expansion rates under each combination described above. Maximal expansion rate was obtained with IL-2 and rapamycin (23.7 fold expansion +/−12.7 at day 7 and 89 fold expansion +/−32 at day 14). The 3 molecules cocktail significantly reduced initial nTreg proliferation (2.3 fold expansion +/−0.57, day 7). However, after 2 weeks of culture nTreg were nevertheless amplified 13.7 +/−5.9 fold, only three times less than in the presence of IL-2 alone (38.7 +/−4.1) (Figure 4B). We next sought to optimize the proliferation rate of nTreg cells in the presence of RAV. The aforementioned expansion rates were obtained upon culture in U-bottom wells. We thus cultured the cells in flat bottom wells and could obtained better expansion rates as nTreg cells in the presence of RAV amplified 37 +/−18 fold after 2 weeks of culture (*n* = 3, Figure 4B).

Thus, the combination of rapamycin, vorinostat and azacytidin (RAV) is suitable for the optimal expansion of nTreg maintaining high expression of FOXP3 in the presence of IL-2.

### Combination of azacytidin, vorinostat, rapamycin and IL-2 preserves nTreg cells suppressive function *in vitro* and *in vivo*

**Figure 5:**
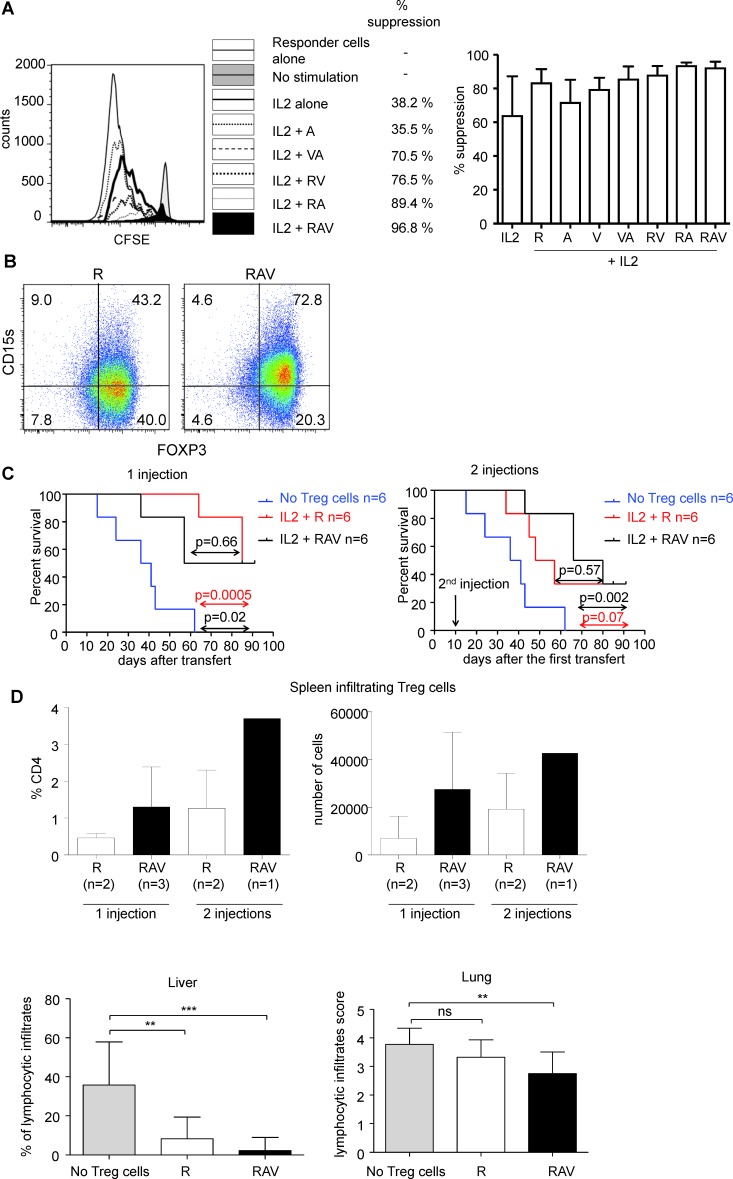
RAV combination preserves *in vitro* and *in vivo* suppressive nTreg cells function **A.** nTreg cells cultured for 14 days in days in the presence of indicated combinations of R, A and V molecules were co-cultured with autologous CD25^−^CD45RA^+^CD4^+^T responder cells labeled with CFSE in the presence of plate-bound anti-CD3 (0.5 microg/mL) and autologous irradiated B cells and monocytes. CFSE dilution was analyzed after 96 hours of culture on gated CD4^+^CFSE^+^ T cells. % of suppression are indicated. One experiment representative out of 4 independent experiments is shown (left). Mean % suppression by nTreg cells cultured for 12-14 days in the presence of indicated combinations of R, A and V molecules (*n* = 4). Error bars indicate s.d (right). **B.** Expanding nTreg cells upregulate CD15s *in vitro* in the presence of RAV combination. nTreg cells were FACS isolated and cultured for 14 days in the presence of anti-CD3/CD28 beads, IL-2 and R (left) or in the presence of R, A and V (right). Cells were then analyzed for the expression of CD15s and FOXP3. Data are representative of 3 independent experiments. **C.** nTreg cells (2×10^6 cells) cultured for 14 days under RAV regimen (*n* = 6, black line) or rapamycin (*n* = 6, red line) were co-transferred with 2×10^6 autologous PBMCs in NSG mice (top left). Expanded nTreg cells with RAV (*n* = 6, black line) or rapamycin (*n* = 6, red line) were re-injected 10 days after the cotransfer in other mice (top right). Survival of the mice were compared to NSG mice injected with 2×10^6 PBMCs alone (blue line, *n* = 6). Comparisons (purple arrow for R *vs* RAV comparison, red for R vs PBMCs alone and black for RAV vs PBMCs alone) were made using a Log-rank (Mantel-Cox) Test. *p* value < 0.05 is considered significant (top). Proportions of FOXP3 expressing cells among CD4^+^ T cells and absolute counts of FOXP3^+^CD4^+^ T cells in the spleen of surviving mice at day 91. Of note, cells from 1 spleen from the rapamycin 1 injection group and from 1 spleen of the RAV 2 injections group could not be analyzed because these spleens were highly fibrotic (bottom). **D.** Histopathological analysis of liver and lung of mice injected with PBMCs, PBMCs and nTreg cells expanded in the presence of rapamycin alone (R) or rapamycin associated with vorinostat and azacytidin (RAV). Statistical comparisons were made using unpaired t test. *vs* < 0.05 are considered significant. **p < 0.01 ***p < 0.001 ns: not significant.

We then verified that nTreg cells expanded under the three drug regimen retained their suppressive capacities *in vitro*. As shown, nTreg cells expanded for 10 to 14 days in the presence of IL-2 and the combination of IL-2, rapamycin, vorinostat and azacytidin potently suppress autologous responder T cell proliferation. Of note, nTreg cells expanded in the presence of each molecule alone or in the presence of RA or VA combinations showed less potent suppression than nTreg cells expanded under the three drug regimen while nTreg cells expanded in the presence of RA showed equivalent suppressive capacities (Figure 5A).

We have recently shown that surface expression of CD15s (sialyl Lewis x) identifies circulating highly suppressive FOXP3^high^ effector Treg cells. While naïve Treg cells do not express CD15s when isolated ex vivo and while only a minority of the latter upregulated CD15s after activation even in the presence of rapamycin [21], we could observe that most of expanding nTreg cells expressed CD15s in the presence of RAV combination (Figure 5B). This result further indicates that the RAV combination enables the expansion of CD4^+^ T cells with phenotypic and functional properties that correspond to those observed on ex vivo isolated effector Treg cells i.e. they display high levels of FOXP3, are CD15s^+^ and are highly suppressive *in vitro*.

Finally, we evaluated the suppressive capacity of nTreg cells expanded in the presence of RAV combination *in vivo* as their capacity to delay xeno GVHD in NOD common gamma chain (−/−) SCID mice (NSG) mice. Irradiated NSG mice develop severe GVH disease within 3 weeks after the transfer of 2 million human PBMCs. In comparison, when the same amount of human PBMCs was co-transferred with 2 million nTreg cells expanded for 14 days under RAV condition *in vitro*, we observed that GVHD was significantly delayed. All mice injected with PBMCs alone (*n* = 6) were dead 2 months after the infusion of PBMCs while at least half of the mice co-injected with PBMCs and expanded nTreg cells were alive. Some of surviving mice kept increasing weight in both ramamycin alone and RAV groups (Supplementary Figure 3). The survival of mice co-transferred with PBMCs and nTreg cells cultured with RAV (RAV mice, *n* = 6) was comparable to the survival of mice co-injected with PBMCs and nTregs cells cultured with rapamycin (R mice, *n* = 6), indicating that RAV regimen is at least as efficient as rapamycin alone for the expansion of *in vivo* suppressive nTreg cells. Of note, the number of FOXP3^+^ Treg cells in the spleen of surviving mice at day 90 was significantly higher in RAV mice (Figure 5C).

Finally, we evaluated whether a second injection of expanded nTreg cells 10 days after the first injection could lead to better survival. No difference was observed in terms of survival in mice injected twice with nTreg cells expanded with RAV (*n* = 6) or rapamycin alone (*n* = 6) when compared to mice with a single injection (*p* = 0.23 for the comparision of 1 infusion of nTreg cells cultured with rapamycin *vs* 2 infusions and *p* = 0.87 for the corresponding comparison for nTreg cells cultured with the RAV regimen (Figure 5C).

While the overall survival was not statistically different when comparing R mice and RAV mice, we could observe that the lymphocytic infiltrates were milder in the liver at day 10 and in the lung at day 90 when Treg cells were expanded in the presence of RAV (Figure 5D).

Altogether, these results indicate that a 10 to 14 days culture with high dose IL-2 and the combination of rapamycin, histone deacetylase inhibitor vorinostat and DNA methyltransferase inhibitor azacytidin is optimal for the expansion of human CD45RA^+^ naïve Treg cells with sustained high expression of FOXP3 and potent suppression capacities *in vitro* and *in vivo*.

## DISCUSSION

Because Treg cells are required for the maintenance of self tolerance *in vivo*, Treg-based cell therapy has been proposed as a novel strategy to treat autoimmune diseases or GVHD and to promote tolerance to allografts [2]. Because Treg cells represent a minority of CD4^+^ T cells, expansion steps are mandatory in order to infuse enough cells in patients [10, 14]. During the last years, several groups have proposed different strategies to expand human Treg cells *in vitro* leading to deceptive results in term of FOXP3 expression levels at the end of the process [22, 28, 29].

Given that FOXP3 expressing CD4^+^ T cells are heterogeneous, inefficient Treg cells expansion can be the result of an inadequate choice of FOXP3^+^ subset(s) as starting material. Because human Treg cells have initially been described as CD4^+^CD25^high^ cells, initial expansion strategies focused on the latter and did not take into account more advanced definitions distinguishing between CD45RA^+^ and CD45RA^−^ cells and between FOXP3^high^ and FOXP3^low^ cells [2, 9, 30]. The weak expansion rate, purity and intensity of expression of FOXP3 in expanded CD4^+^CD25^high^ cells can be explained by (1) the presence within CD25^high^ cells of FOXP3^high^ cells with poor expansion potentiality, (2) the low proportion of nTreg cells and (3) the presence of contaminating FOXP3^low^ non Treg cells. Other groups have focused on CD4^+^CD25^+^CD127^low^/^−^ cells because this phenotypic definition indeed encompasses all FOXP3 expressing CD4^+^ T cells [31, 32]. However, the impossibility to expand a homogeneous FOXP3^bright^ population starting from sorted CD4^+^CD25^+^CD127^low/−^ underlines the inclusion of numerous FOXP3^low^ non Tregs cells in this definition [33]. Indeed, most of the latter cannot maintain FOXP3 expression even in the presence of high dose IL-2 (Figures 1 and 2). Of note, a subset of FOXP3^low^ non Treg cells displayed high levels of FOXP3 upon expansion indicating that some FOXP3^low^ CD45RA^−^ non Treg cells may have eTreg differentiation potentiality (Figures 1 and 2). This finding further indicates that FOXP3^+^ non Treg cells are heterogenous, not only in their ability to produce cytokines [20, 34] but also in their capacity to adopt an eTreg profile.

Hofmann et al. have therefore proposed that CD45RA^+^ nTreg cells would represent better candidates for *in vitro* expansion [28]. They nevertheless observed that repetitive stimulation of nTreg cells *in vitro* led to the FOXP3 downregulation [18]. We also observed that nTreg cells as well eTreg cells could lose FOXP3 expression, even in the absence of repetitive TCR engagement (Figure 1).

Here we show that the presently considered optimal Treg expansion protocol, that is, high dose IL-2 combined to rapamycin, a molecule widely known to enhance Treg purity by eliminating non Treg cells [22], was insufficient for the induction and/or maintenance of high FOXP3 levels in expanded nTreg cells (Figure 2).

Our objective was therefore to determine which other combination of drug candidates could allow expansion of pure Treg populations with preserved immunosuppressive function. Combination of IL-2 with rapamycin, histone deacetylase inhibitor vorinostat and DNA methyltransferase inhibitor azacytidin met these requirements. Indeed, we showed that nTreg cells expanded under this regimen expressed CD15s [21] and were potently suppressive *in vitro*. We have recently shown that CD15s is a surface marker specific for potently suppressive Treg cells with high expression of FOXP3. CD15s is involved in the transmigration of cells toward endothelial cells through interactions with P- and E-selectins [35]. Therefore, isolation of expanding Treg cells bearing CD15s using anti-CD15s antibodies may not be suitable for cell therapy as anti-CD15s antibodies might impair their migration to target organs by neutralizing CD15s and thus prevent suppression in tissues *in vivo*.

Because it is not yet established that *in vitro* suppression is equivalent to *in vivo* Treg mediated suppression, we conducted experiments in NSG mice and could confirm that nTreg cells expanded in that way could indeed prevent GVH disease, although not completely (Figure 5). This is in line with previous results showing that purified Treg cells could not prevent xeno GVH lethality in the NSG mice [36]. Because the widely accepted reference regimen for the *in vitro* expansion of nTreg cells is based on the use of rapamycin, we compared the efficiency of nTreg cells expanded in the presence of RAV with those expanded in the presence of rapamycin in the prevention of GVH disease in mice. Because the survival rate of mice in both groups was similar, we conclude that RAV regimen is at least as efficient as rapamycin for the expansion of *in vivo* suppressive nTreg cells. Moreover, we observed a higher number of residual nTreg cells in the spleen of mice injected with nTreg cells expanded in the presence of RAV, milder lymphocytic infiltrates in the liver and the lung, indicating that cell therapy strategies based of the infusion of nTreg cells with RAV combination may have a better long term efficacy than nTregs expanded with rapamycin alone.

Expansion of pure Tregs populations free of contaminating non-Treg cells is presently obtained at the cost of culture yield. Indeed, the expansion rate of nTreg cells under this optimized condition remains relatively modest compared to other published strategies [28, 29]. We believe that this does not represent a limitation for clinical application since clinical grade strategies to isolate a large number of leukocytes through leukapheresis are already widely used. From 2.10^7^ to 10^8^ sorted cells, it can be expected to expand from 8 × 10^8^ to 4 × 10^9^ FOXP3^high^ nTreg cells (i.e. 1.3×10^7^ to 6×10^8^ nTregs per kg for a standard 60 kg weight) after 14 days of culture.

We also showed that multiple sequential infusions of expanded nTreg cells were not necessary to control GHVD as a second infusion of expanded nTreg cells did not improve the survival of mice. This result brings further evidence that expanded nTreg cells are suited for the prevention of GVHD and may not be accurate to ameliorate an ongoing disease.

Since the present protocol was optimized using clinical grade reagents (X vivo culture media, AB human serum, cGMP antiCD3/anti-CD28 beads, clinical grade IL-2, rapamycin, azacytidin and vorinostat), RAV-expanded naïve Treg cells could be rapidly available for clinical evaluation. Whether infusion of nTreg cells cultured using this condition is safe in healthy volunteers and in patients is still yet to be determined in controlled clinical prospective studies.

## MATERIAL AND METHODS

### Cell isolation and flow cytometry

PBMCs were isolated through Ficoll gradient separation from freshly drawn blood. CD4^+^T cells were first magnetically isolated using a CD4 T cell separation kit (Miltenyi) and subsequently surface stained using a combination of flurochrome-conjugated mAbs: anti-CD4-PErCP 5.5, anti-CD25-PE, anti-CD127-AF647 and anti-CD45RA-FITC obtained from BD bioscience. CD25^−^CD45RA^+^CD4^+^, FOXP3^low^ non Treg, naïve FOXP3^low^CD45RA^+^ and effector FOXP3^high^CD45RA^−^ Treg cells were flow isolated from PBMCs following flow isolation according to the gating strategy we validated previously using a FACSAria (BD bioscience) [20] (Supplementary Figure 1).

Expanded cells were counted and flow analyzed every week for the expression of FOXP3 and Ki-67 after fixation and permeabilization (eBioscience) on a FACSCanto (BDBioscience) cytometer and analyzed using FlowJo software (Treestar). Cells were stained with PerCP-anti CD4, PE-anti FOXP3 (259D/C7 clone), AF647-CD15s (CSLEX1) and FITC-anti Ki67-mAb (BDbiosciences). Because FOXP3 expression cut-off that differentiates positive cells from negative cells is different in cultured cells compared to ex vivo isolated cells, we also cultured CD4^+^CD25^−^CD45RA^+^ cells as control cells that were used to the determinate the right threshold for FOXP3 expression in expanded Treg cells and non Treg cells.

### Cell culture

Isolated cells were immediately distributed into U or flat bottom well for culture and expansion. To analyze the effect of tested molecules or combination of molecules, 10 to 30 *10^3^ evaluated cells were collected per U bottom. Cells were cultured in conditions suitable for clinical application that is, X-vivo 15 media, (Lonza) with 5% AB serum (Invitrogen Lifetech) and supplemendted with 2mM L-glutamin, 1mM sodium pyruvate,1% non essential amino acid MEM, 100U/mL penicillin, 100 μg/ml streptomycin and amphotericin B (all from Gibco) anti-CD3/anti-CD28 coated Treg expander beads (Invitrogen Lifetech). Cells evaluated for expansion were first polyclonally stimulated using anti-CD3/anti-CD28 beads (concentration: 40 000 beads/microL; 2 microL/well) in the presence of 300 IU/mL IL-2 (Miltenyi Biotec) in culture media in the presence of a combination of Rapamycin (Sigma-Aldrich) diluted in culture medium (1 microg/mL), Azacytidin (Vidaza^®^ from Celgene) first diluted in PBS and then in culture medium (5 microM) and Vorinostat (Selleck chem) first diluted in DMSO and then in culture medium (1 microM). 300 to 1000 IU/mL IL-2 was added every 3-4 days. Cells were cultured for 7, 14 or 21 days in U or flat bottom wells. Cells were counted and analyzed for FOXP3 expression at day 7 and day 14.

### *In vitro* suppressive assays

Expanded cells were assessed for suppressive function between 10 and 14 days of culture. 1 × 10^4^ CFSE (1μM Invitrogen)-labeled responder CD25^−^CD45RA^+^CD4^+^ T cells were cocultured with 1 × 10^4^ unlabeled cells assessed for their suppressive capacity and 1 × 10^5^ irradiated autologous accessory cells containing B cells and monocytes. Cells were stimulated with 0.5 μg/mL plate-bound anti-CD3 (OKT3 mAb) in 96-well round-bottom plate in RPMI medium supplemented with 10% fetal bovin serum (Bio West), 2mM L-glutamin, 1mM sodium pyruvate,1% non essential amino acid MEM, 100U/mL penicillin, 100 μg/ml streptomycin and amphotericin B (all from Gibco). Proliferation of CFSE-labeled cells was assessed by flow cytometry after 84-90 hr of culture. Percent suppression was calculated as follows: [1 - (number of proliferating CFSE diluting responder cells in the presence of suppressor cells at a 1 to 1 ratio / number of proliferating responder cells when cultured alone)] x 100.

### Mice

NOD.Cg-Prkdc^scid^ Il2rg^tm1Wjl^/SzJ (NSG) (stock ≠005557, The Jackson Laboratory) mice were bred in our own animal facilities in Specific Pathogen-Free conditions (accreditation number from the Veterinary services: A75-13-10) with an enriched fat regime and addition of Bactrim in drinking water every other week. The colony was regularly checked for γ-c deficiency by PCR according to the Jackson Laboratory protocol. All procedures were approved by the Regional Ethical Commitee on Animal Experimentation.

### Induction of xeno-Graft *vs*. Host disease in NSG mice

Young adult NSG female mice (11 weeks old) were irradiated (2 Gy) and received 2×10^6^ PBMCs from a healthy donor with or without 2×10^6^ autologous Treg cells cultured in IL-2 + RAV conditions or rapamycin alone for 14 days. Some mice received a second infusion of 10^6^ expanded cells 10 days after the first injection. Animals were euthanatized if their weight dropped below 80% of their initial weight or when the weight was stabilized or increased 90 days after the injection without signs of GVH disease.

### Histological analysis

Liver and lungs were embedded in OCT and immediately frozen in liquid nitrogen. Organs were further sectioned (6μm thick) for hematoxylin-eosin staining. Slides were coded without reference to prior treatment and examined in a blinded fashion for quantification. For liver, localized infiltrates were numbered and normalized with vessel number. For lungs, degree of infiltrate was scored according to a scale going to 0 ( = no infiltrate) to 5 ( = covered field infiltrate 100%). Histological images were acquired with an Olympus CK2 microscope (Shinjuku, Tokyo, Japan) plus Moticam 2300 camera, and analysed with Motic Software (Motic Asia, Hong Kong).

## SUPPLEMENTARY MATERIALS FIGURES


