# Supplementary material for: Combination of IL-2, rapamycin, DNA methyltransferase and histone deacetylase inhibitors for the expansion of human regulatory T cells
Source: Oncotarget. 2016 Jul 28;8(62):104733–44. doi: 10.18632/oncotarget.10914 (PMC5739596; doi:10.18632/oncotarget.10914)
Supplement: Supplementary file 1 [file oncotarget-08-104733-s001.pdf]

Combination of IL-2, rapamycin, DNA methyltransferase and histone deacetylase inhibitors for the expansion of human regulatory T cells

Supplementary Material

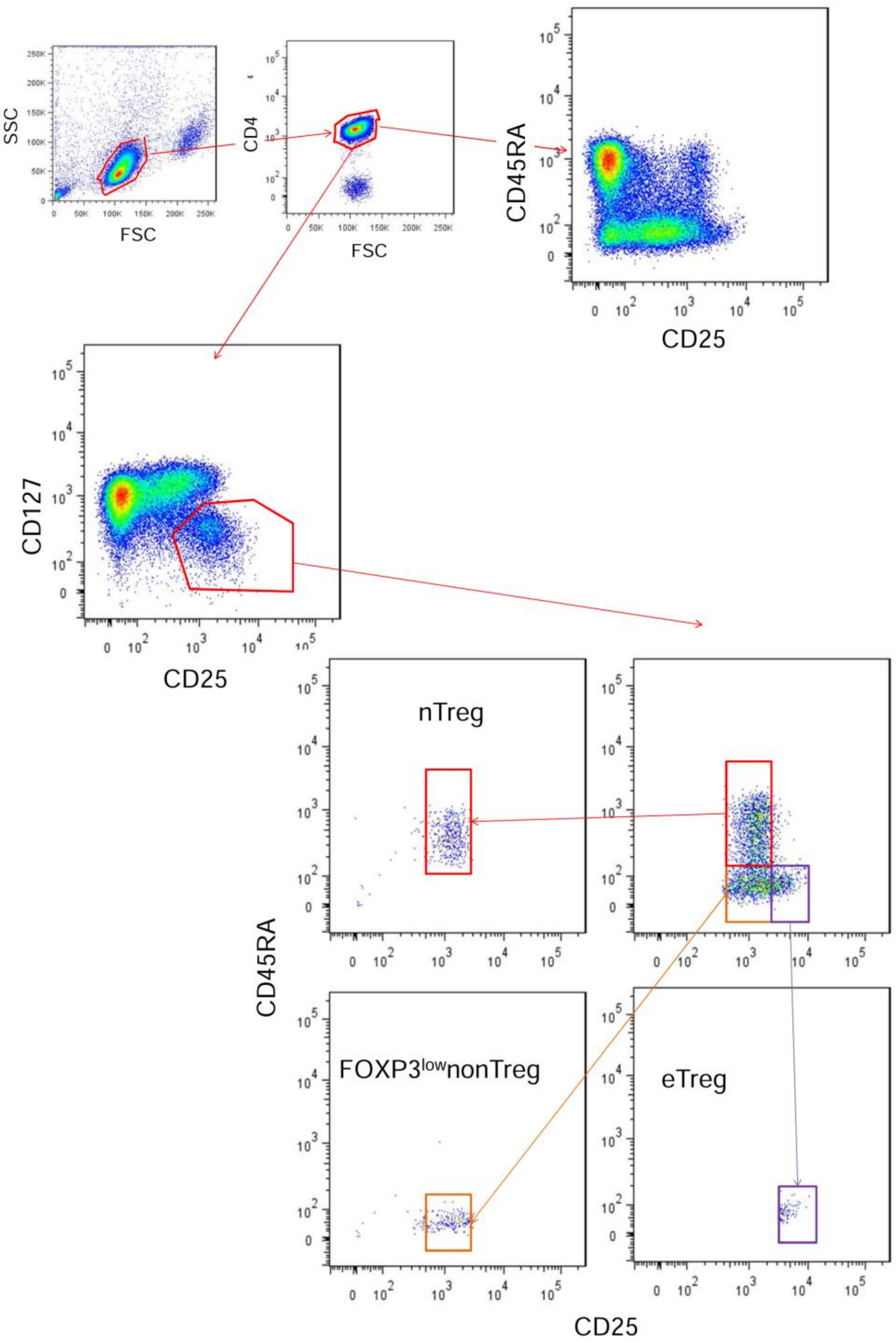

Supplementary Figure 1: Gating strategy for the isolation of FoxP3 expressing $CD4^+$ T cell subsets.

PBMCs obtained from healthy donors were stained with anti-CD4, antiCD25, anti-CD127 and CD45RA, magnetic bead sorted for CD4 and flow separated using a FACS Aria (BD bioscience).

A

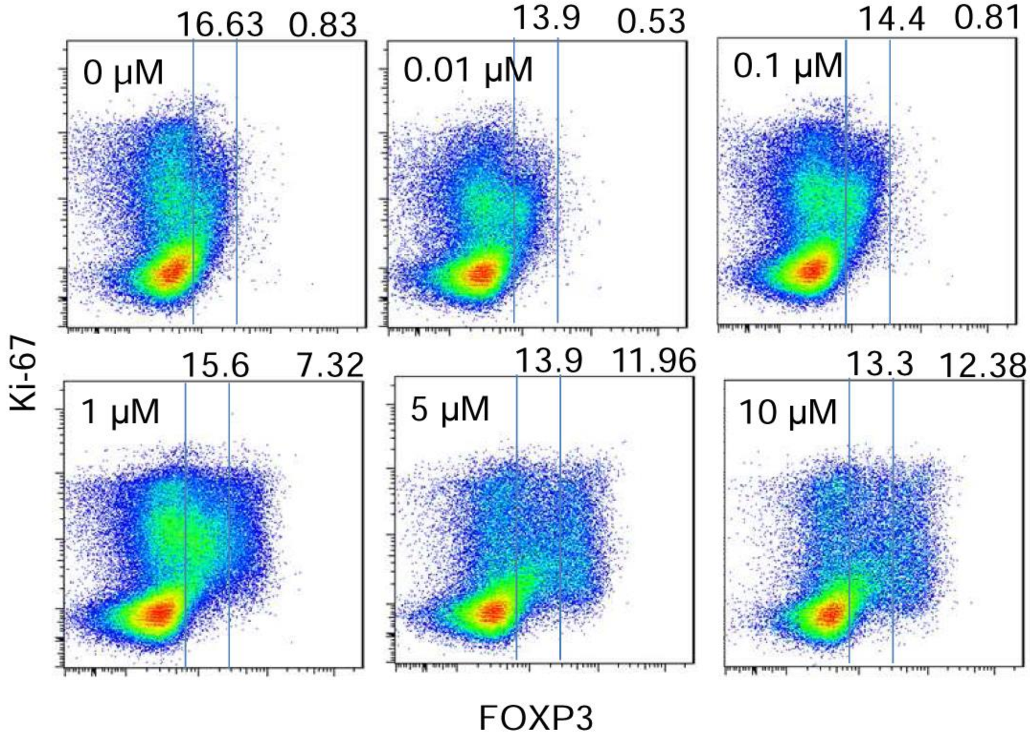
16.63
0.83
0 µM
13.9
0.53
0.01 µM
14.4
0.81
0.1 µM
15.6
7.32
1 µM
13.9
11.96
5 µM
13.3
12.38
10 µM
Ki-67
FOXP3

B

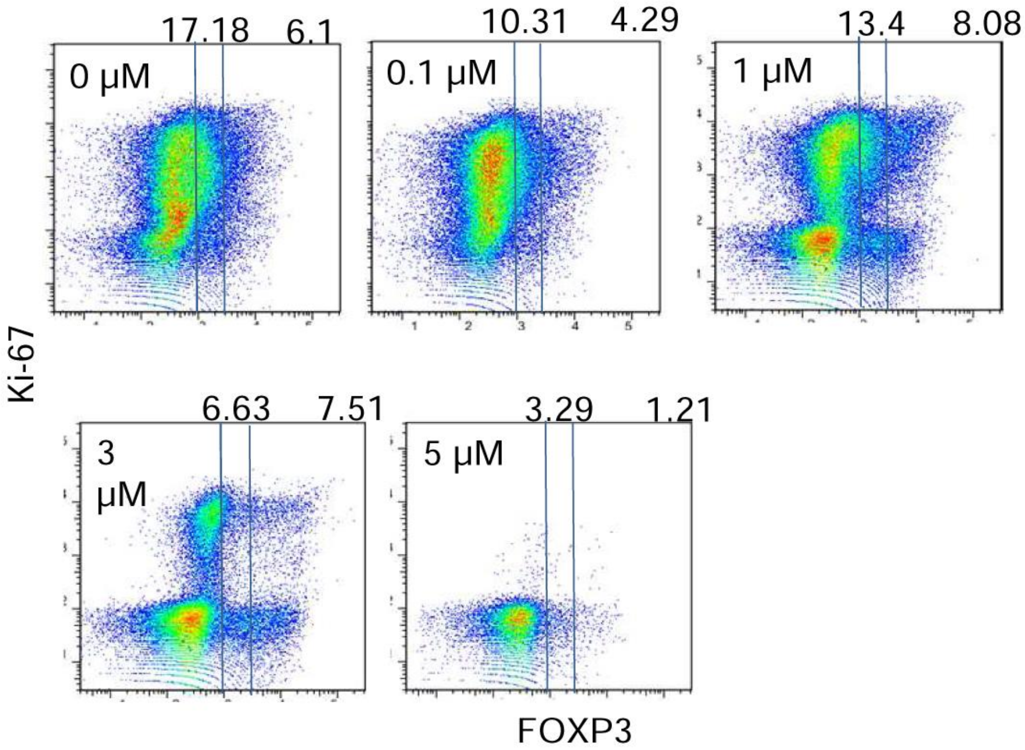
17.18
6.1
0 µM
10.31
4.29
0.1 µM
13.4
8.08
1 µM
6.63
7.51
3 µM
3.29
1.21
5 µM
Ki-67
FOXP3

Supplementary Figure 2: DNA methyl transferase and histone deacetylase inhibitors enhance FoxP3 expression in activated $CD4^+$ T cells

Whole PBMCs from healthy donors were cultured in the presence of IL-2, anti-CD3/CD28 beads and indicated concentrations of azacytidin (A) or vorinostat (B). FoxP3 and Ki-67 levels on gated $CD4^+$ T cells were FACS analyzed after 48-72 hours of culture. Proportions of $CD4^+$ T cells with low and high expression of FoxP3 are indicated. Data shown are representative of 2 independent experiments. Proportions (%) of FoxP3low and FoxP3high cells are indicated in the top of each panel.

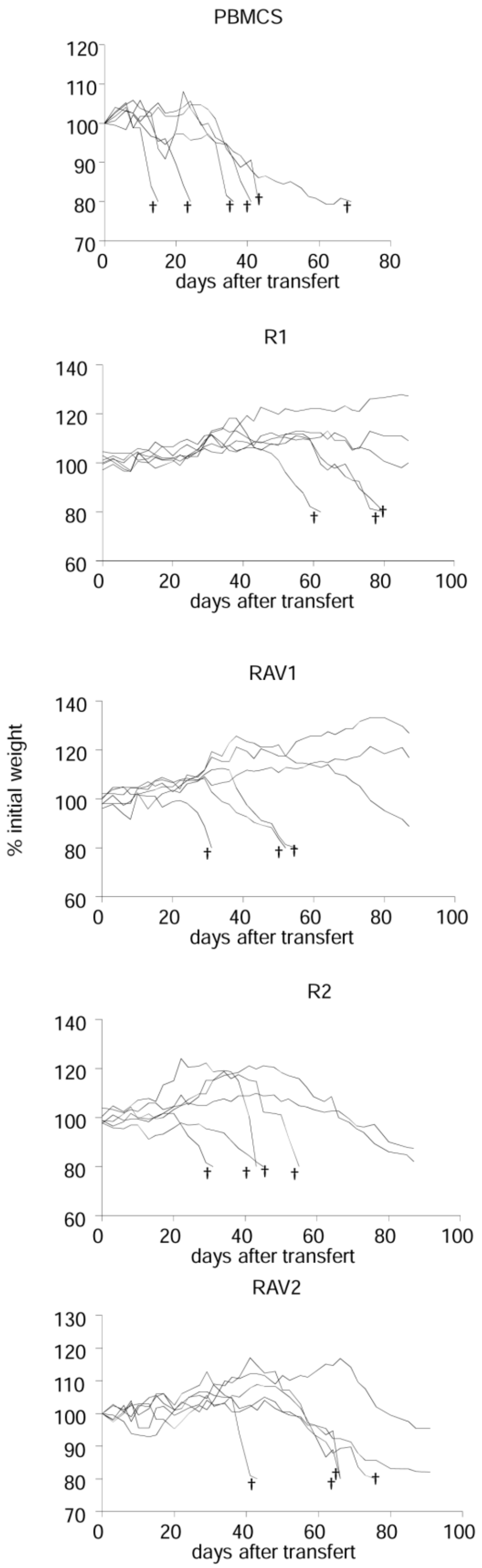
PBMCS
120
110
100
90
80
70
0
20
40
60
80
days after transfert
R1
140
120
100
80
60
0
20
40
60
80
100
days after transfert
RAV1
% initial weight
140
120
100
80
60
0
20
40
60
80
100
days after transfert
R2
140
120
100
80
60
0
20
40
60
80
100
days after transfert
RAV2
130
120
110
100
90
80
70
0
20
40
60
80
100
days after transfert

Supplementary Figure 3: RAV combination preserves *in vivo* suppressive nTreg function

nTreg cells (2x10^6 cells) cultured for 14 days under RAV regimen (n=6, RAV 1 injection) or rapamycin (n=6, R 1 injection) were co-transferred with 2x10^6 autologous PBMCs in NSG mice (top left). Expanded nTreg cells with RAV (n=6, RAV 2 injections) or rapamycin (n=6, R 2 injections) were re-injected 10 days after the cotransfer in other mice (top right). Survival of NSG mice injected with 2x10^6 PBMCs alone is also shown (n=6, PBMCs alone).

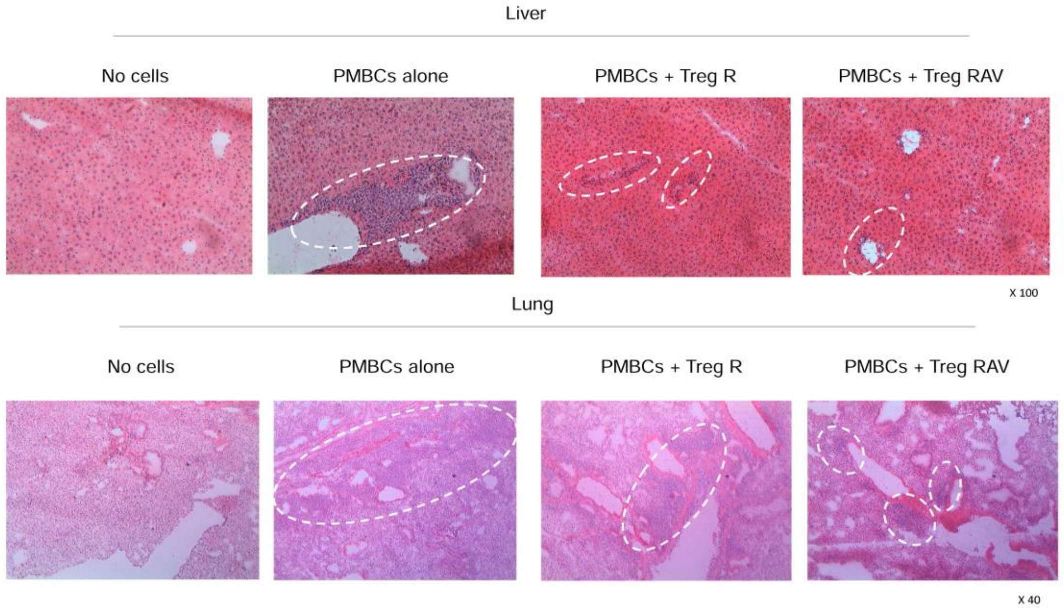

Supplementary Figure 4: Histopathological analysis of liver and lung of mice injected with PBMCs, PBMCs and nTreg cells expanded in the presence of rapamycin alone or rapamycin (R) associated with vorinostat and azacytidin (RAV). Broken line circles surround lymphocytic infiltrates.
